# Enhanced Migratory Waterfowl Distribution Modeling by Inclusion of Depth to Water Table Data

**DOI:** 10.1371/journal.pone.0030142

**Published:** 2012-01-17

**Authors:** Betty J. Kreakie, Ying Fan, Timothy H. Keitt

**Affiliations:** 1 Section of Integrative Biology, University of Texas at Austin, Austin, Texas, United States of America; 2 Department of Earth and Planetary Sciences, Rutgers University, Piscataway, New Jersey, United States of America,; Biodiversity Insitute of Ontario - University of Guelph, Canada

## Abstract

In addition to being used as a tool for ecological understanding, management and conservation of migratory waterfowl rely heavily on distribution models; yet these models have poor accuracy when compared to models of other bird groups. The goal of this study is to offer methods to enhance our ability to accurately model the spatial distributions of six migratory waterfowl species. This goal is accomplished by creating models based on species-specific annual cycles and introducing a depth to water table (DWT) data set. The DWT data set, a wetland proxy, is a simulated long-term measure of the point either at or below the surface where climate and geological/topographic water fluxes balance. For species occurrences, the USGS' banding bird data for six relatively common species was used. Distribution models are constructed using Random Forest and MaxEnt. Random Forest classification of habitat and non-habitat provided a measure of DWT variable importance, which indicated that DWT is as important, and often more important, to model accuracy as temperature, precipitation, elevation, and an alternative wetland measure. MaxEnt models that included DWT in addition to traditional predictor variables had a considerable increase in classification accuracy. Also, MaxEnt models created with DWT often had higher accuracy when compared with models created with an alternative measure of wetland habitat. By comparing maps of predicted probability of occurrence and response curves, it is possible to explore how different species respond to water table depth and how a species responds in different seasons. The results of this analysis also illustrate that, as expected, all waterfowl species are tightly affiliated with shallow water table habitat. However, this study illustrates that the intensity of affiliation is not constant between seasons for a species, nor is it consistent between species.

## Introduction

Species distribution models, especially for migratory waterfowl, are employed as a tool in diverse areas of investigation and application [1]. For example, distribution models have been used to help explore how the interactions between migratory waterfowl and landscape factors will impact the spread of diseases [2]–[4]. These approaches are used to understand how migratory birds might influence the health of the ecosystem through which they move [5], [6]. The economics of waterfowl hunting draw on distribution modeling to optimize long term success of these game species and thus the sport [7], [8]. Perhaps the field that most heavily relies on distribution modeling is those that attempt to forecast how waterfowl will respond to anthropogenic disturbances, such as climate change [9], [10].

Given the dependency of waterfowl research and management on distribution modeling, it is critical that these tools be of the highest quality. Yet it has been shown that distribution models for birds that are migratory and have high wetland affinity are less accurate than those models for species that do not have these specific ecological traits [11]. Seasonal changes, in not only spatial location, but also habitat selection, contribute to this decrease in model capacity [12], [13]. Compounding temporal factors affecting model accuracy, waterfowl are reliant on wetland habitats, which are a poorly recorded habitat type [14]. Even though there may be well-mapped modern wetland data available, due to the dynamic and often ephemeral nature of wetlands, this data will most likely be insufficient for time series analysis [15], [16].

The goal of this study is to offer new strategies that will enhance distribution modeling of migratory waterfowl throughout their entire annual cycle. For each species included in our study, distribution models were constructed for each portion of the annual cycle (i.e. fall, winter/non-breeding, spring, and summer/breeding). The delineation of these events is species specific, which allows for reciprocal species specific variation in predictor variables. Even though species specific distributions were created, we utilized the availability of the community data to more accurately generate pseudo-absences when necessary [17]. Additionally, this study introduces a novel data set to use as a predictor variable in distribution modeling of wetland species. This wetland data set, depth to water table (DWT), is a simulated long-term measure of the point either at or below the surface where climate and geological/topographic water fluxes balance [18].

The inclusion of wetland proxy data is a common technique used to attempt to overcome the difficulties of modeling species with high wetland affinity [1], [19]. These measures range from fine scale research with direct measure of wetland quality [20], through large scale research that incorporates watershed-level hydrological modeling [21], [22], to potentially global-scale relatively fine grain classified satellite imagery [23]. When habitat variables have been included in distribution modeling, they are based on current classifications and not model-based prediction. In addition to the advances already made to account for wetland influence of species distribution, the inclusion of DWT data in distribution modeling provides numerous advantages to this field. The DWT data are process-driven, and allow us to investigate the underlying hydrologic drivers that may influence habitat selection. Furthermore, the DWT has a large spatial extent (nearly global) and fine resolution (approximately 270 m). The inclusion of this process-driven wetland proxy data will potentially allow us to overcome the shortcomings of forecasting future spatial distributions of countless wetland species with other approaches (such as climate envelopes) [24], [25].

The presented research is intended to augment the approaches used to construct distribution models for migratory waterfowl. We assembled distribution models for species-specific annual cycles. This allows us to assess spatial distributions throughout the entire annual cycle, not just focusing on one portion, while adjusting for differences in timing between species. Furthermore, a novel data set, DWT, is introduced and shown to be an important predictor variable of migratory waterfowl habitat. These data are calculations of hydrological balances between climate and geology, which will allow for more mechanistic approaches to constructing distribution models for wetland species. Overall, the strategies presented in this research will enhance and improve distribution modeling of migratory waterfowl, and in turn allow for better management and conservation of these species.

## Methods

### Species Data

The United State Geological Survey's (USGS') Banding Bird Laboratory (BBL) game bird dataset was used as the source of species presence data. Established in 1902, the BBL is a long-term monitoring project with over three million waterfowl encounter records [26]. The data record the incidence of banding and band-recovery events within 10-minute bins of latitude and longitude. Most bands are reported by hunters and are thus terminal encounters. Each banding location and all subsequent encounter locations were treated as a known presence for that species. Owing to uncertainty in exact encounter locations, the BBL data only provide locations at 10-minute resolution (approximately 16 km); however this resolution is sufficiently fine given the broad spatial extent of our analysis. We restricted our analysis to banding and encounter events from January 1, 1990 through December 31, 1999, which we considered to be enough time to capture the main trend of defining the spatial distribution.

Six species were included in the study: American black duck (*Anas rubripes*), blue-winged teal (*Anas discors*), Canada goose (*Branta canadensis*), mallard (*Anas platyrhynchos*), northern pintail (*Anas acuta*), and wood duck (*Aix sponsa*) (Table 1). These species were chosen because they have the highest numbers of encounters, and also had adequate sampling in all portions of the annual cycle. All six species are in the family *Anatidae*, which are deemed typical waterfowl [27], [28]. Canada goose is in the subfamily *Anserinae* (geese and swan), while all other species are in the subfamily *Anatinae* (dabbling ducks).

**Table 1:** Season specific MaxEnt AUC scores for each study species.

Fall	n	Base+DWT	Base+PW	DWT only
ABDUC	762	0.9202	0.914	0.7274
BWTE	1403	0.8203	0.821	0.7444
CAGO	3223	0.8193	0.8172	0.6658
MALL	5959	0.7332	0.7304	0.6253
NOPI	314	0.8585	0.8668	0.5881
WODU	1307	0.8529	0.8494	0.7844

Winter				
ABDUC	765	0.9304	0.9254	0.7649
BWTE	180	0.9544	0.9527	0.9153
CAGO	4149	0.7373	0.7354	0.6217
MALL	4198	0.7886	0.7814	0.6712
NOPI	1458	0.8064	0.8036	0.7368
WODU	1633	0.9251	0.9229	0.8249

Spring				
ABDUC	18	0.9544	0.9542	0.7464
BWTE	18	0.844	0.8659	0.6666
CAGO	225	0.7684	0.7525	0.634
MALL	151	0.7902	0.7856	0.5968
NOPI	27	0.8331	0.8659	0.646
WODU	126	0.8784	0.8825	0.7584

Summer				
ABDUC	27	0.9208	0.9244	0.6942
BWTE	44	0.8294	0.826	0.5696
CAGO	2255	0.7493	0.7433	0.6671
MALL	651	0.8045	0.8034	0.5888
NOPI	33	0.866	0.8799	0.6048
WODU	2606	0.8447	0.8401	0.7009

The “base” variables are temperature, precipitation, and elevation. Models were constructed using the two different measures of wetland: average water table depth (DWT) from dynamically-driven hydrology model and percent wetland (PW) based on land cover classification. See Figure 3 for species abbreviations.

**Figure 1:**
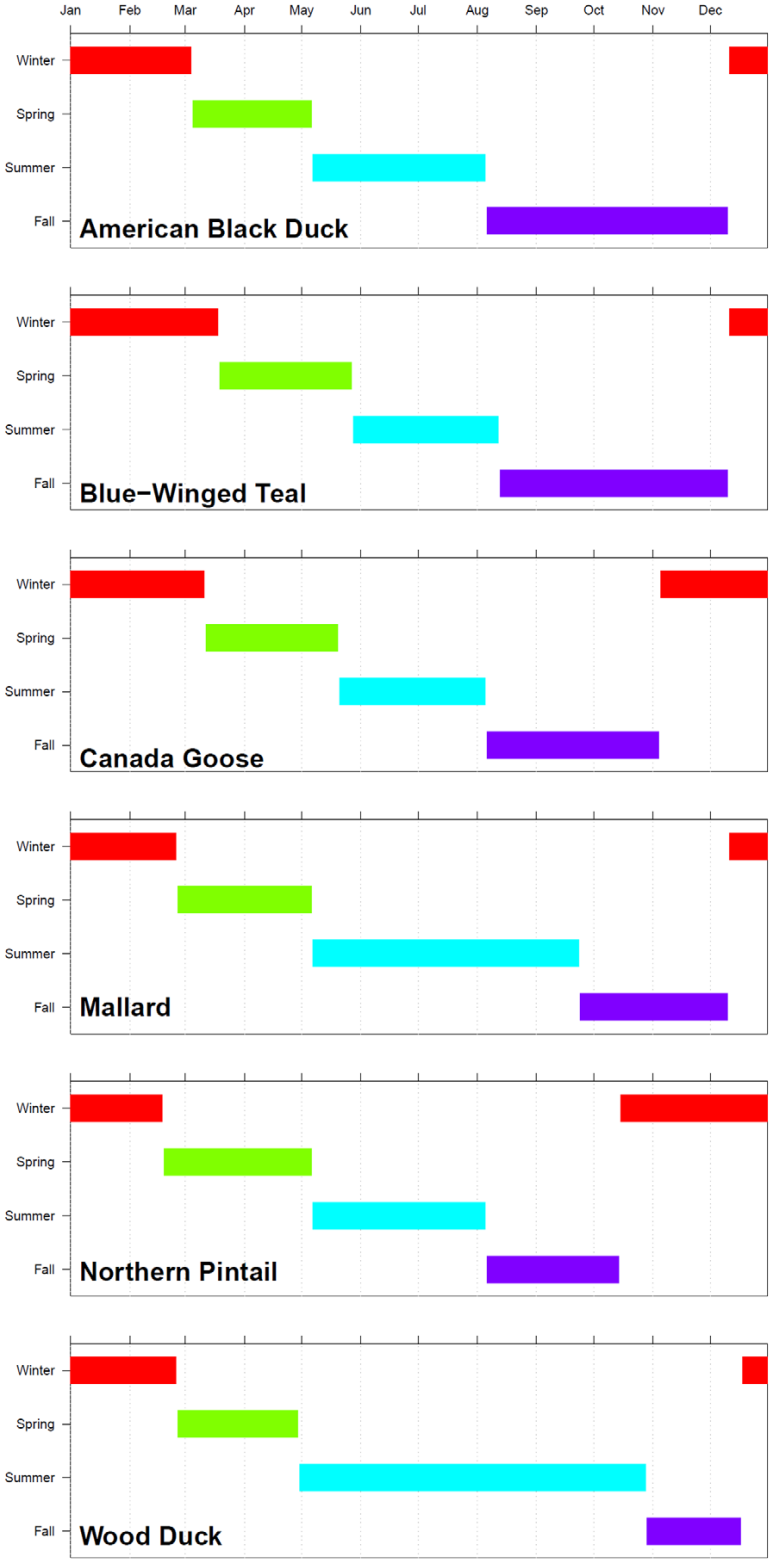
Barplot of annual cycle timing for study species.

Investigation of seasonal differences in distribution and habitat use required delineating the four major components of the annual cycle. BBL data was used to determine which times of the year individuals were traveling the greatest average daily distance, and these peaks in velocity were labeled as fall and spring migration. Because of the potential for confounding inter-season movement, our analysis was restricted to the mean daily traveled distance of those records where bands were recovered within 30 days of being banded. For each within-30-days recovery, the total great circle distance traveled, calculated using the “geosphere” package in R [29], was divided by the total number of days between banding and recovery. Fall and spring migration were delineated by locating peaks in the average weekly distance traveled, and summer and winter were dated according to the appropriate intermediate seasons. These results were compared to our initial dates established by natural histories [30]–[32] and the dates were adjusted when clear migration signals were present (Figure 1).

### Environmental Data

All predictor variables were resampled, throughout the contiguous United States study extent, to agree with the BBL data grid. Average seasonal temperature, average seasonal precipitation, and elevation were used in all distribution models. Average monthly precipitation and monthly average temperature data were obtained from the Prism Climate Group [33], originally a 2.5-minute (approximately 4 km) resolution. We used the 3-second (approximately 90 m) Shuttle Radar Topography Mission (SRTM) Elevation Data Set. In addition to the three previously mentioned variables, one of two different wetland measures were included. A derived variable of percent classified wetland was created from the 2001 National Land Cover Database (NLCD) 30-meter data [34]. The 2001 NLCD data is a land use-land cover classification of satellite, Landstat imagery. Models were built with the inclusion of the percent NLCD wetland as a point of comparison for the models build with the DWT data.

**Figure 2:**
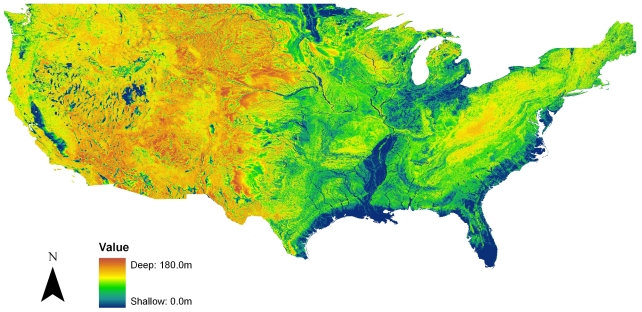
Map of the simulated equilibrium water table depth for the contiguous US [**18**]. The values illustrate the depth in meters below the surface where the simulated water table is located.

The DWT data layer is a simulated data set that reliably predicts the location of natural wetlands (Figure 2) [18], [35]. The depth to water table is determined by finding the long-term stable solution of the balance between the climate-driven fluxes (precipitation and evapotranspiration) and geologic/topographic water fluxes (riverine and groundwater movement) balance. Initially, the water table was set at the surface and at each time step the modeled DWT was recalculated based on water inputs or outputs. The model was allowed to run until the water table for each cell (9-second resolution) was stable (less than 1 mm change). The DWT model was validated using 500,000+ USGS field observations of water table depth from 1927–2005; the mean of the residuals (simulated DWT – observed DWT) is 0.443 m. Fan and Miguez-Macho [18] further tested the ability of the data to locate wetlands on the landscape. They found a strong correlation (0.8469) between field-mapped wetlands and the simulated data thresholded to 1.0 m water table depth. There is a -0.36 correlation between the DWT data and NLCD percent wetland data, which was used as an alternative measure of wetland habitat for this study. The DWT data were obtained directly from Fan and Miguez-Macho, and the referenced manuscripts provide in-depth details on model development and validation [18], [35].

### Statistical Analysis and Distribution Modeling

Random Forest was used to robustly gauge variable importance [36]. Random Forest is a technique that fits multiple classification trees (specifically 1000 trees) [37], [38]. Each unique classification created is considered a “class,” and every time this class is created it receives a vote. The class with the highest number of votes is selected as the final output. The individual trees are built by recursively resampling the data into two groups: approximate 63% training and 37% test. The test data provides a means to test not only model accuracy, but also variable importance. We used the mean decrease in accuracy, which is the normalized difference between classification accuracy and the accuracy when the variable values have been randomly permuted. Higher mean decrease in accuracy indicates that a variable is more important to the accuracy of the classification. All Random Forest analyses used the ‘randomForest’ package in R [39].

Because the BBL data is a presence-only data set, background points (or pseudo-absences) were created for Random Forest. Background points for each species and season were identified as known locations of other study species where the focal species was absent. This approach to generating background points ameliorates the bias of uneven sampling effort [40].

MaxEnt was selected for creating the species distribution models; it is a maximum entropy approach specifically for presences-only data [41], [42]. It was implemented in MaxEnt 3.3.2 software package, and model set according to Phillips and Dudik [42]. Models for each season for all species were run a total of 100 times, randomizing the 70-30 training-test split of the data and the location of the background points.

Model performance of MaxEnt was measured using Area-Under-the-Curve (AUC) scores. AUC is the measure of the area under a receiver operating characteristic (ROC) curve; specifically plotting the rate of true positive classification to false positive [43], [44]. AUC typically ranges from 0.5 (essentially random) to 1.0 (perfect fit). In addition to MaxEnt and Random Forest, we create GLM models for all species in each season. These results were consistent with the MaxEnt and Random Forest results, and therefore are not presented here.

The predicted probability of occurrence maps and model response curves from the MaxEnt models are presented. MaxEnt models are presented due to the fact that this method was created specifically for presence-only data, and its ability to better address the sampling bias of the BBL data [45]. Also, only models for the winter portion of the annual cycle are presented. Winter was selected due to the relatively high sample intensity and resulting model accuracy. All other seasons' results are available in the supporting information.

## Results

**Figure 3:**
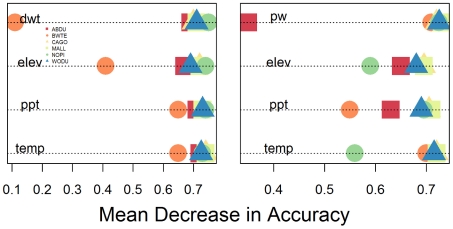
Plot of variable importance measure from Random Forest. Variable importance is measured in mean decrease in accuracy, which is the decrease in accuracy of a classification after the variable has been randomly permuted. A higher mean decrease in accuracy means the variable contributes more to the accuracy of the classification. The abbreviations are as follows: ABDU (American black duck), BWTE (blue-winged teal), CAGO (Canada goose), MALL (mallard), NOPI (northern pintail), WODU (wood duck), temp (temperature), ppt (precipitation), elev (elevation), dwt (depth to water table), and pw (NLCD's percent wetland).

Random Forest was used to measure variable importance on the accuracy of classification of presences and absences. More specifically, it was used to determine how important DWT was to the overall model and how it compared to the other predictor variables (see Figure 3 for winter results and Figure S1 for all other seasons). Depth to water table was consistently as important as the other customary predictor variables: temperature, precipitation, and elevation. Also, DWT's importance was comparable to the importance of NLCD percent wetland for Random Forest models. The importance of DWT varied by species; with it being least important for the classification of blue-winged teal in the winter (mean decrease in accuracy = 0.097). During winter, DWT was most important for northern pintail (mean decrease in accuracy = 0.75). Comparing the importance throughout the annual cycle, DWT had the highest importance values for the spring (ranging from 1.4 for wood duck to 2.28 for American black duck).

MaxEnt models created with a wetland variable (DWT or NLCD percent wetland) had considerably higher AUC score than those models (from here forward referred to as “base models”) created with only temperature, precipitation, and elevation. When the AUC of MaxEnt models for each species were directly compared between base with DWT and base with percent wetland, 11 MaxEnt models build with DWT had a higher AUC than percent wetland model for that species (table 1). Six MaxEnt models had no statistical difference between AUC scores, and 4 MaxEnt base and percent wetland models had higher AUC scores than the reciprocal base and DWT model. Nine of the 24 MaxEnt models built with only DWT as predictor variable had an AUC over 0.7.

**Figure 4:**
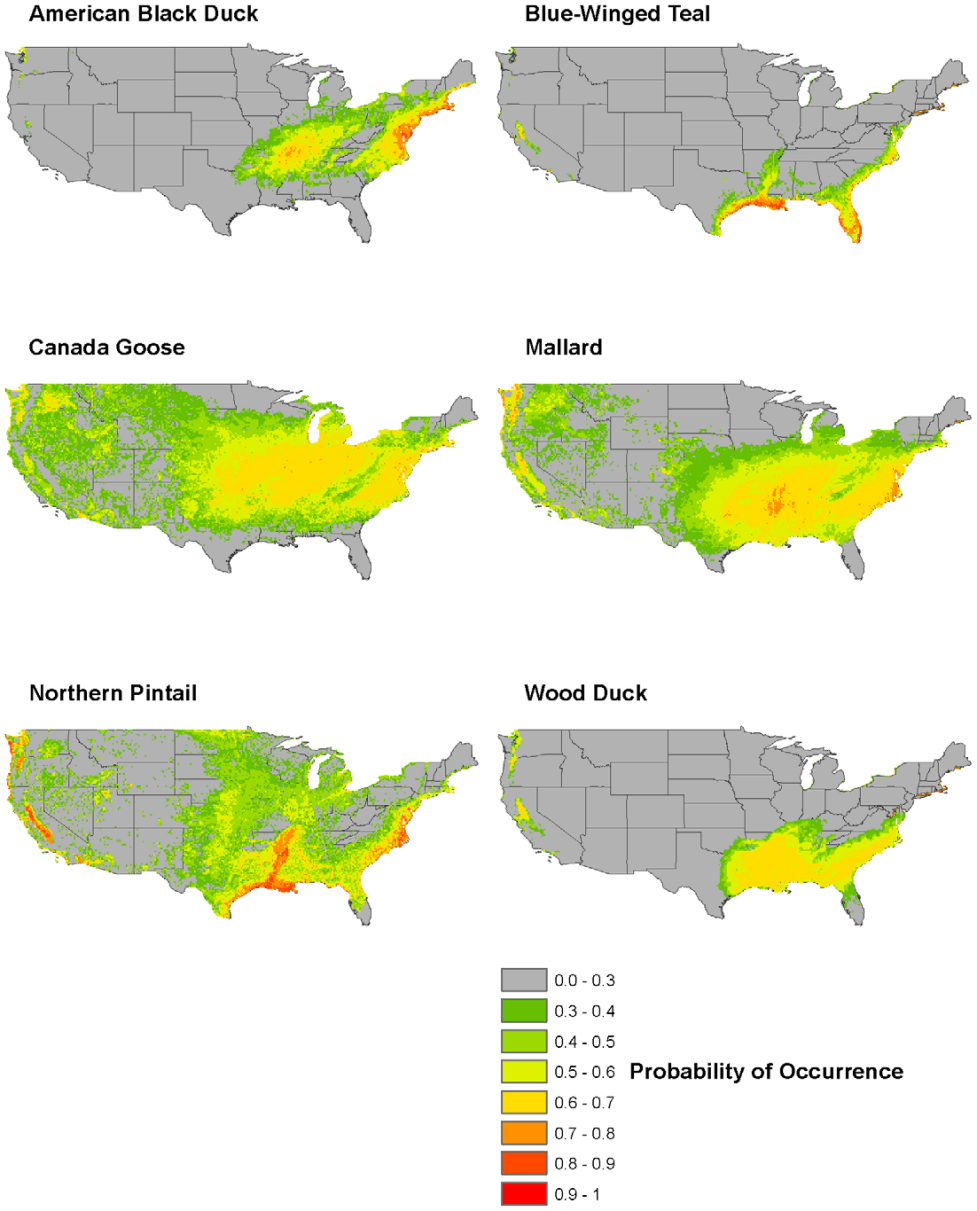
Maps of predicted probability of occurrence for all study species' winter habitat. Predictions were created using MaxEnt with 100% of known presence locations to increase accuracy of the visual representation. Temperature, precipitation, elevation, and water table depth were the predicted variables used to construct the probability surfaces.

The MaxEnt winter predictions, created with base predictor variables and DWT, for all species are presented in Figure 4 (see Figures S2, S3, S4 for all other seasons). Predictions are in line with the fact that all species should be centered in the southern portions of their ranges. The highest predicted values for blue-winged teal, northern pintail, and wood duck are along the southern portion of the east coast and the Gulf of Mexico coast up the Mississippi River. Canada goose and mallard, to a lesser degree, have large areas of mid-range predicted probability of occurrence in the central portion of the United States. American black duck's predictions are focused in the northeastern portion of the country, while avoiding the peaks of the Appalachian Mountains. All species have some moderate predictions along the west coast, especially in the Central Valley in California.

**Figure 5:**
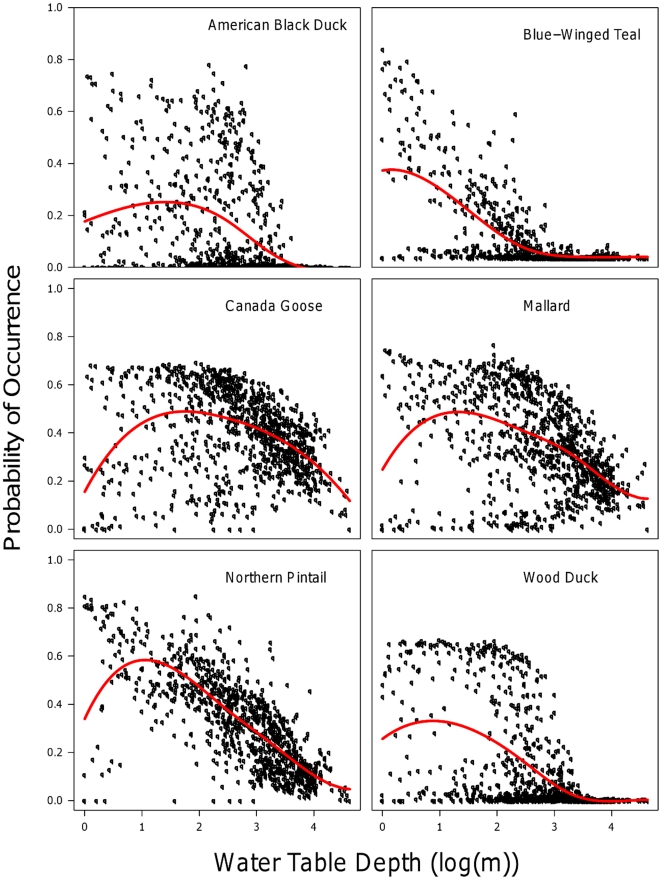
Plot of relationship between water table depth (m) and occurrence probability for species in winter. The plots were constructed by selecting 1,000 random points from the predicted probability of occurrence surface. The red curve is a smoothing spline fit to the mean of the data points, and is meant only to visually illustrate the trend of the data and the upper threshold of DWT.

For each of the study species in the winter, the relationship between DWT and MaxEnt predicted probability of occurrence is presented in Figure 5 (all other response curves are provided in Figures S5, S6, S7). The distribution of each species is skewed towards the shallow water tables. Canada goose and mallard's distribution are less skewed to the left than the other species; they have a more gradual decrease in predicted occurrence as the water table becomes deeper. Northern pintail has the highest peak at 0.6 at the shallowest water table level.

## Discussion

The foremost goal of this study was to present strategies that would enhance our ability to create accurate distribution models of migratory and wetland species. By modeling distributions based on species-specific annual cycles and introducing a novel data set, we were able to successfully accomplish this goal. All species, in all four portions of the annual cycle, had MaxEnt models (base variables plus DWT) with AUC scores greater than 0.7. Additionally, we were able to show that the DWT data set consistently contributed to the distribution models of these species. This was illustrated, first, by showing that DWT was consistently ranked high in variable importance for Random Forest classifications. Secondly, DWT added to the classification accuracy of MaxEnt models when compared to models created with only temperature, precipitation, and elevation. And finally, the DWT data set performed as well, and often better than, as a standard proxy for wetland habitat, classified satellite imagery.

The DWT data offers more advantages to distribution modeling beyond the increased model performance presented here in this research, most of which are due to the fact that it is a model-derived data set. Most importantly the DWT has the potential to be more than a static measure of wetland habitat. The DWT is a measure of the point where hydrologic, topographic, geologic, and climatic fluxes balance. By predicting how the depth to water table changes according to changes in the environment, such as climate change, it will allow for more mechanistic predictions of how wetland species will respond. This data set avoids many of the biases that are present in the more traditional measures of wetland habitat quality or quantity. For example, if using field delineated wetland maps, especially for studies at the continental scale, there is a concern that all those who did the delineation were using the same definition of a wetland [46]. Additionally for studies of large spatial extent, there are often large gaps in digitally available mapped wetland data. These concerns are also true for classified satellite imagery. Often it is unclear, if what is being classified as wetland is truly wetland on the ground [47].

One concern with the DWT data set for distribution modeling is its accuracy at finer scale. The model from which the DWT data are derived does not for example incorporate detailed data on local water extraction and management. Water levels in many wetlands (and wildlife refuges in particular) are actively managed and therefore are expected to deviate from the DWT data. At the relatively coarse 10-minute scale of this study, these deviations are likely not of great concern as the hydrological model will generally identify low-lying areas where water accumulates. These are the same areas where both managed and unmanaged wetlands will predominantly occur. At finer sub-kilometer scales, the limitations of the modeling approach might however become much more apparent as even small changes in water table could be the difference between wetland habitat and dry ground. We are currently investigating the performance of the DWT data for wetland-species distribution models using fine-scale species occurrence data (Kreakie and Keitt, unpublished data).

In addition to the methodological advantages presented here, this research provides insight into the ecology and behavior of these six species. Each species responds differently to the hydrologic regime, even within the group of waterfowl [48]. By using the response curves (Figure 5), it is possible to quantitatively gauge how each species will respond to the changes in the depth to water table. All six study species have increased predicted probability of occurrence toward shallow depth to water table, but these distributions are not uniform between species. For example, both American black duck and blue-winged teal are more skewed toward the shallow end of depth to water table than Canada goose and mallard. The more uniform predicted probability of occurrence across the range of DWT for mallard and Canada goose could be due to multiple factors. First, this could be due to true behavior of these species. These two are more generalist species, and can often been seen in areas devoid of wetlands (such as golf courses and agricultural fields). Second, this uniform predicted response to DWT could be due to the 10-minute scale of the analysis. Canada goose and mallard prefer to be in wet habitat, but are also fine with wet areas nested within an area of relatively deep DWT (for example, a housing subdivision's retention pond) [49].

We are not only able to compare between species response to DWT, but we can also examine how the predicted response to DWT changes between seasons (Figure 5 and Figure S5, S6, S7). For example, blue-winged teal is tightly constrained to shallow DWT in the winter. However, this predicted behavior changes in the summer/breeding season. We hypothesize that conceivably blue-winged teal is foregoing wetland habitat for drier, and perhaps safer, upland nesting sites. It is also important that this is may be another relic of our 10-minute scale. The breeding area of blue-winged teal is concentrated in the Prairie Pothole region, where there are numerous small wetlands within a relatively dry upland landscape matrix.

This study illustrated that the new process-driven depth to water table data set can be used as a predictor variable in distribution modeling of migratory waterfowl. The depth to water table data set is new and has some important hurdles to overcome, such as how to effectively handle human manipulation of the water table. Yet, despite being in its early period, the future research possibilities are abundant and exciting. To date, forecasting the response of wetland species to climate change has been severally limited due to the dynamic nature of wetlands. This issue becomes compounded when considering migratory species that rely on wetlands for stop-over habitat. It becomes nearly impossible to make predictions about the future of migratory waterfowl and how manage accordingly, when there has been no mechanistic means to forecast key wetland habitat across the entire migration route. The DWT data will allow for us to begin to move beyond these obstacles, and make more vigorous prediction about the future of migratory waterfowl.

## Supporting Information

Figure S1 **Plot of variable importance measure from Random Forest.** Variable importance is measured in mean decrease in accuracy, which is the decrease in accuracy of a classification after the variable has been randomly permuted. A higher mean decrease in accuracy means the variable contributes more to the accuracy of the classification. The abbreviations are as follows: ABDU (American black duck), BWTE (blue-winged teal), CAGO (Canada goose), MALL (mallard), NOPI (northern pintail), WODU (wood duck), temp (temperature), ppt (precipitation), elev (elevation), dwt (depth to water table), and pw (NLCD's percent wetland).
(TIF)

Figure S2 **Maps of predicted probability of occurrence for all study species' fall habitat.** Predictions were created using MaxEnt with 100% of known presence locations to increase accuracy of the visual representation. Temperature, precipitation, elevation, and water table depth were the predicted variables used to construct the probability surfaces.
(TIF)

Figure S3 **Maps of predicted probability of occurrence for all study species' spring habitat.** See Figure S2 for description.
(TIF)

Figure S4 **Maps of predicted probability of occurrence for all study species' summer habitat.** See Figure S2 for description.
(TIF)

Figure S5 **Plot of relationship between water table depth (m) and occurrence probability for species in fall.** The plots were constructed by selecting 1,000 random points from the predicted probability of occurrence surface. The red curve is a smoothing spline fit to the mean of the data points, and meant to illustrate the trend of the data.
(TIF)

Figure S6 **Plot of relationship between water table depth (m) and occurrence probability for species in spring.** See Figure S5 for description.
(TIF)

Figure S7 **Plot of relationship between water table depth (m) and occurrence probability for species in summer.** See Figure S5 for description.
(TIF)
