# Supplementary figures and images for: Enhanced Migratory Waterfowl Distribution Modeling by Inclusion of Depth to Water Table Data
Source: PLoS One. 2012 Jan 17;7(1):e30142. doi: 10.1371/journal.pone.0030142 (PMC3260213; doi:10.1371/journal.pone.0030142)

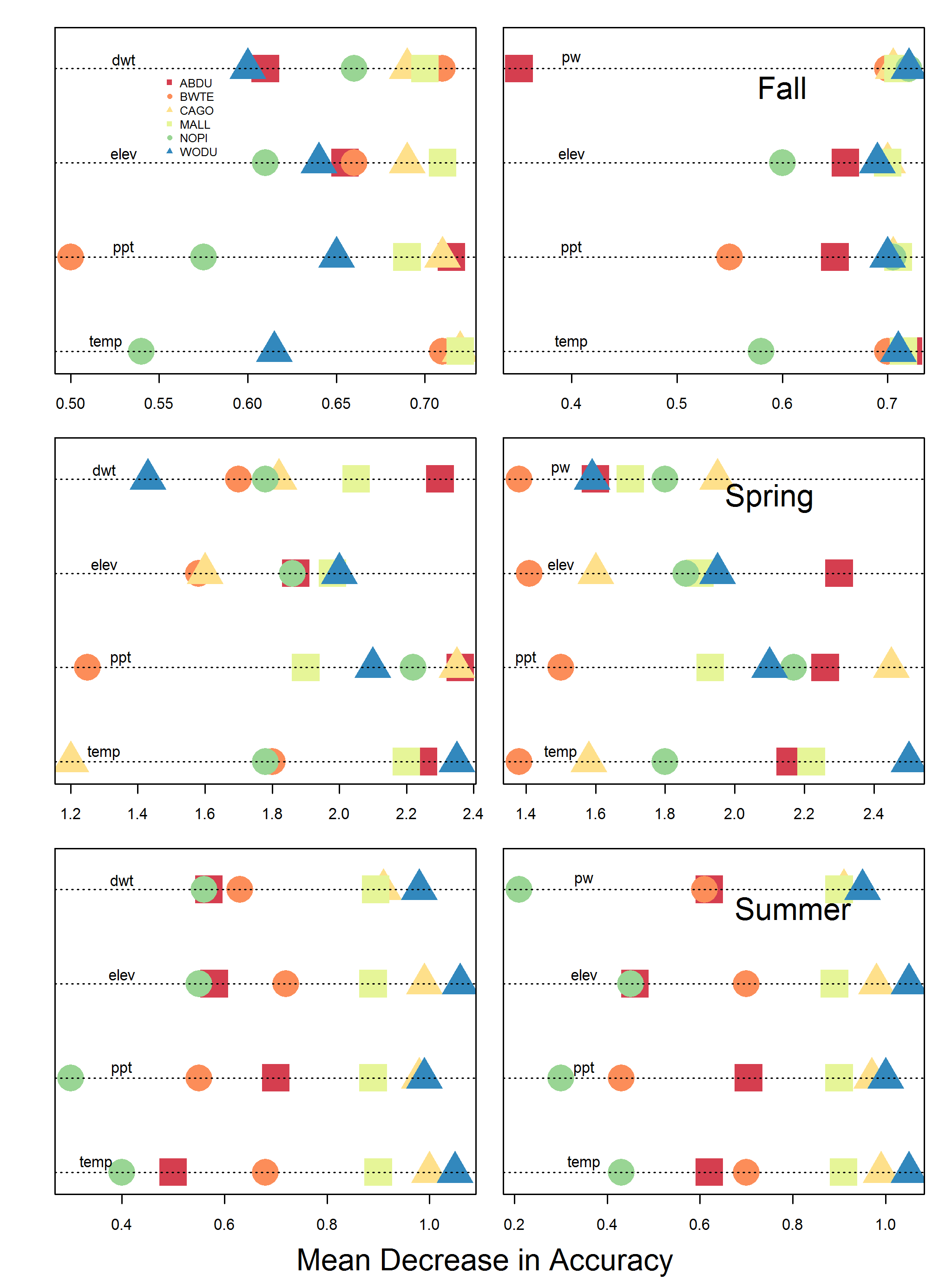

Supplement: Figure S1 — Plot of variable importance measure from Random Forest. Variable importance is measured in mean decrease in accuracy, which is the decrease in accuracy of a classification after the variable has been randomly permuted. A higher mean decrease in accuracy means the variable contributes more to the accuracy of the classification. The abbreviations are as follows: ABDU (American black duck), BWTE (blue-winged teal), CAGO (Canada goose), MALL (mallard), NOPI (northern pintail), WODU (wood duck), temp (temperature), ppt (precipitation), elev (elevation), dwt (depth to water table), and pw (NLCD's percent wetland). (TIF) [file pone.0030142.s001.tif]

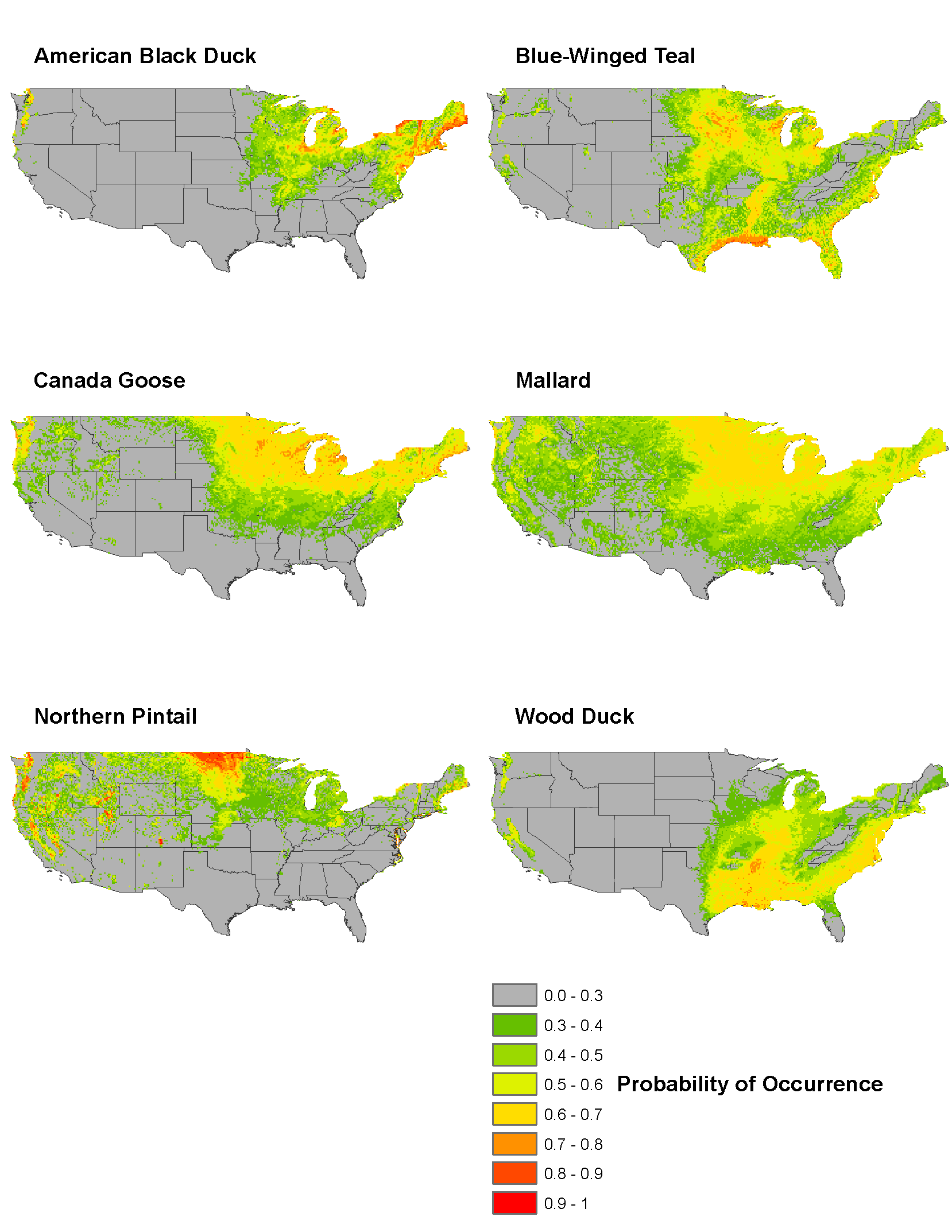

Supplement: Figure S2 — Maps of predicted probability of occurrence for all study species' fall habitat. Predictions were created using MaxEnt with 100% of known presence locations to increase accuracy of the visual representation. Temperature, precipitation, elevation, and water table depth were the predicted variables used to construct the probability surfaces. (TIF) [file pone.0030142.s002.tif]

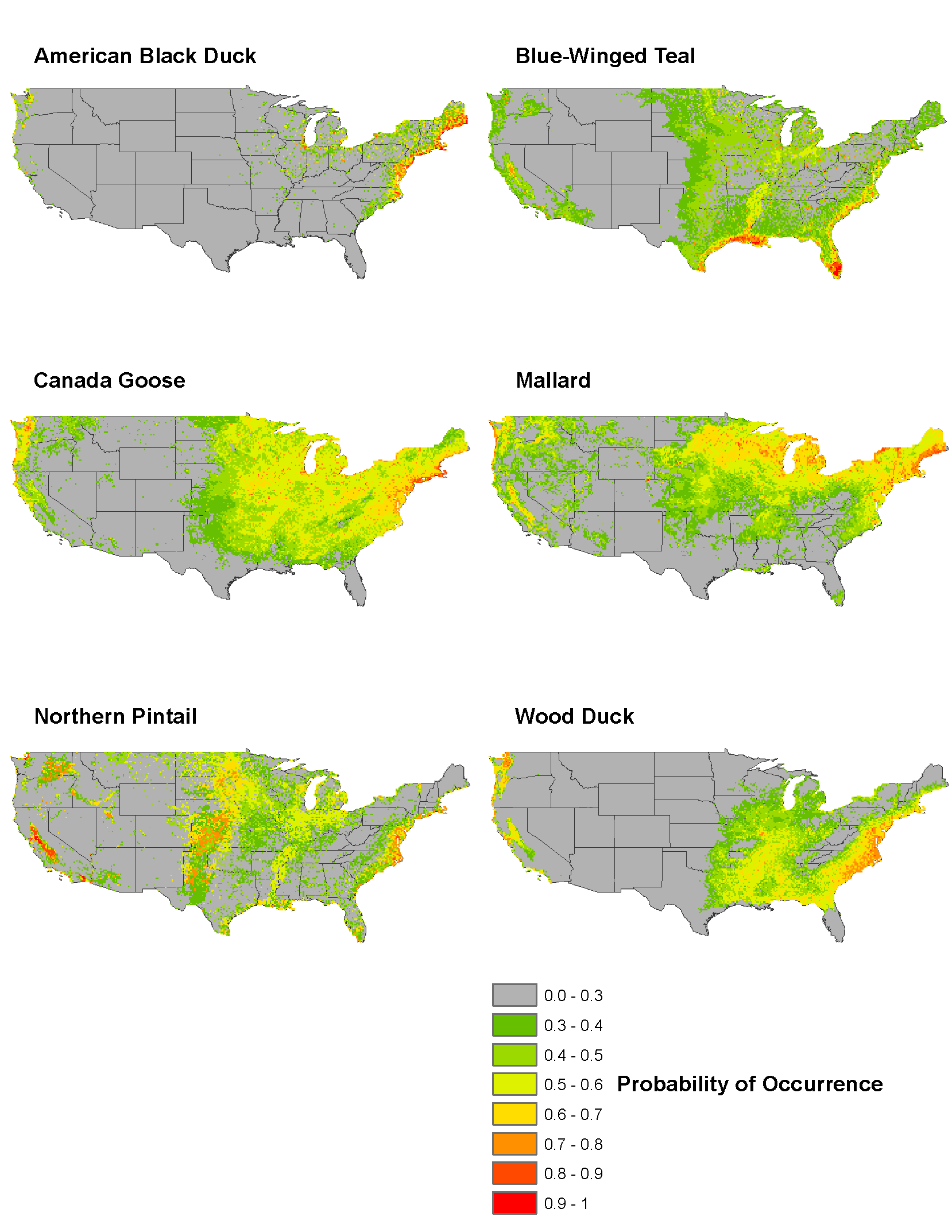

Supplement: Figure S3 — Maps of predicted probability of occurrence for all study species' spring habitat. See Figure S2 for description. (TIF) [file pone.0030142.s003.tif]

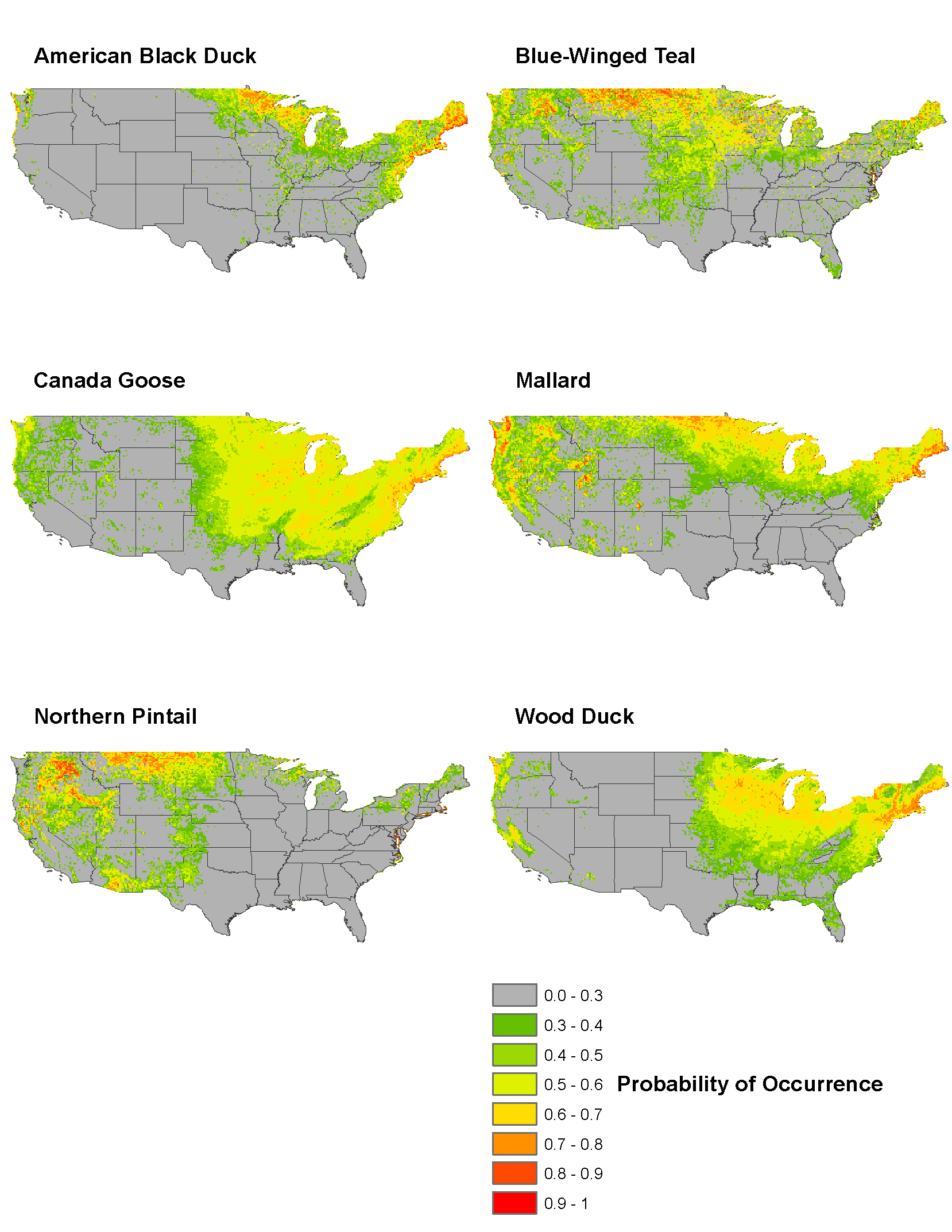

Supplement: Figure S4 — Maps of predicted probability of occurrence for all study species' summer habitat. See Figure S2 for description. (TIF) [file pone.0030142.s004.tif]

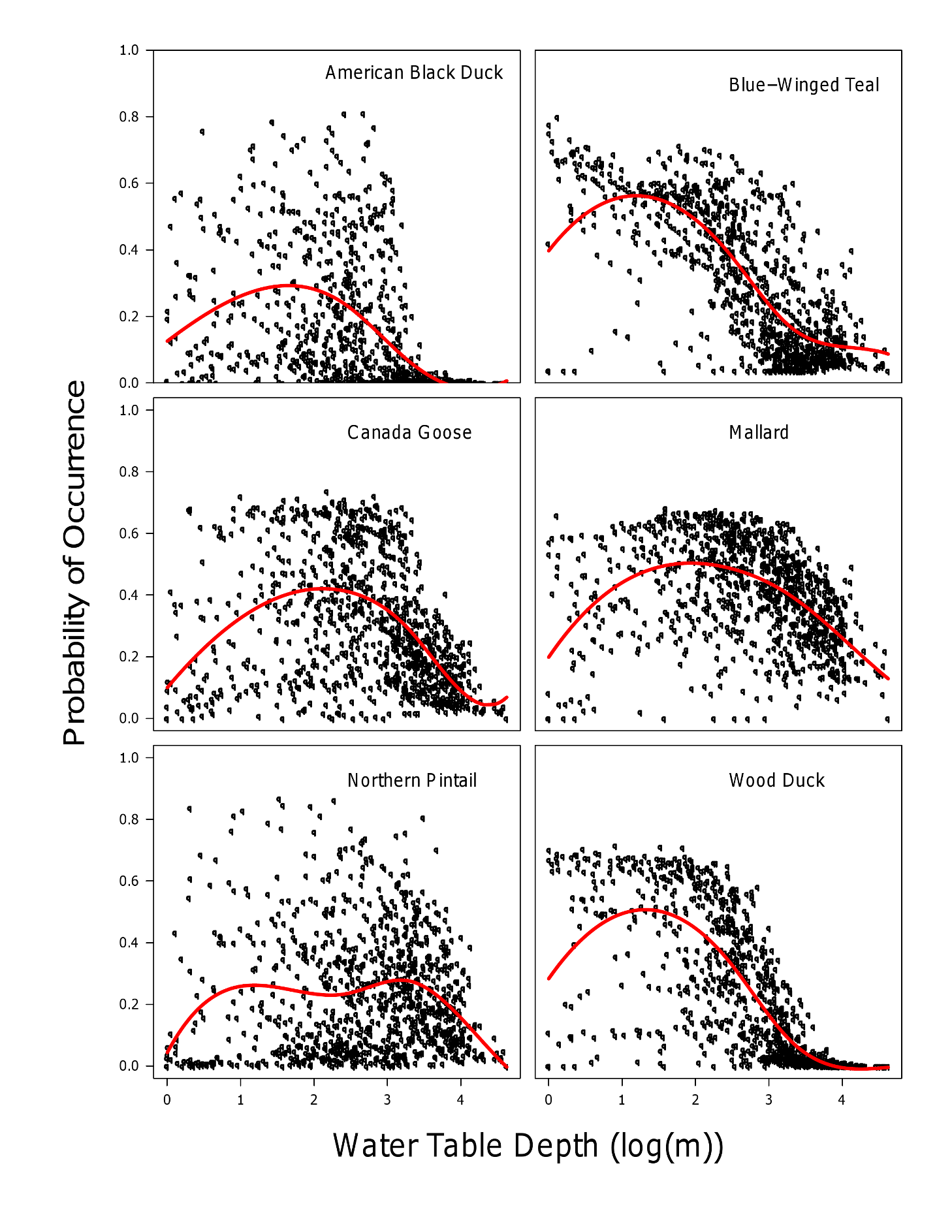

Supplement: Figure S5 — Plot of relationship between water table depth (m) and occurrence probability for species in fall. The plots were constructed by selecting 1,000 random points from the predicted probability of occurrence surface. The red curve is a smoothing spline fit to the mean of the data points, and meant to illustrate the trend of the data. (TIF) [file pone.0030142.s005.tif]

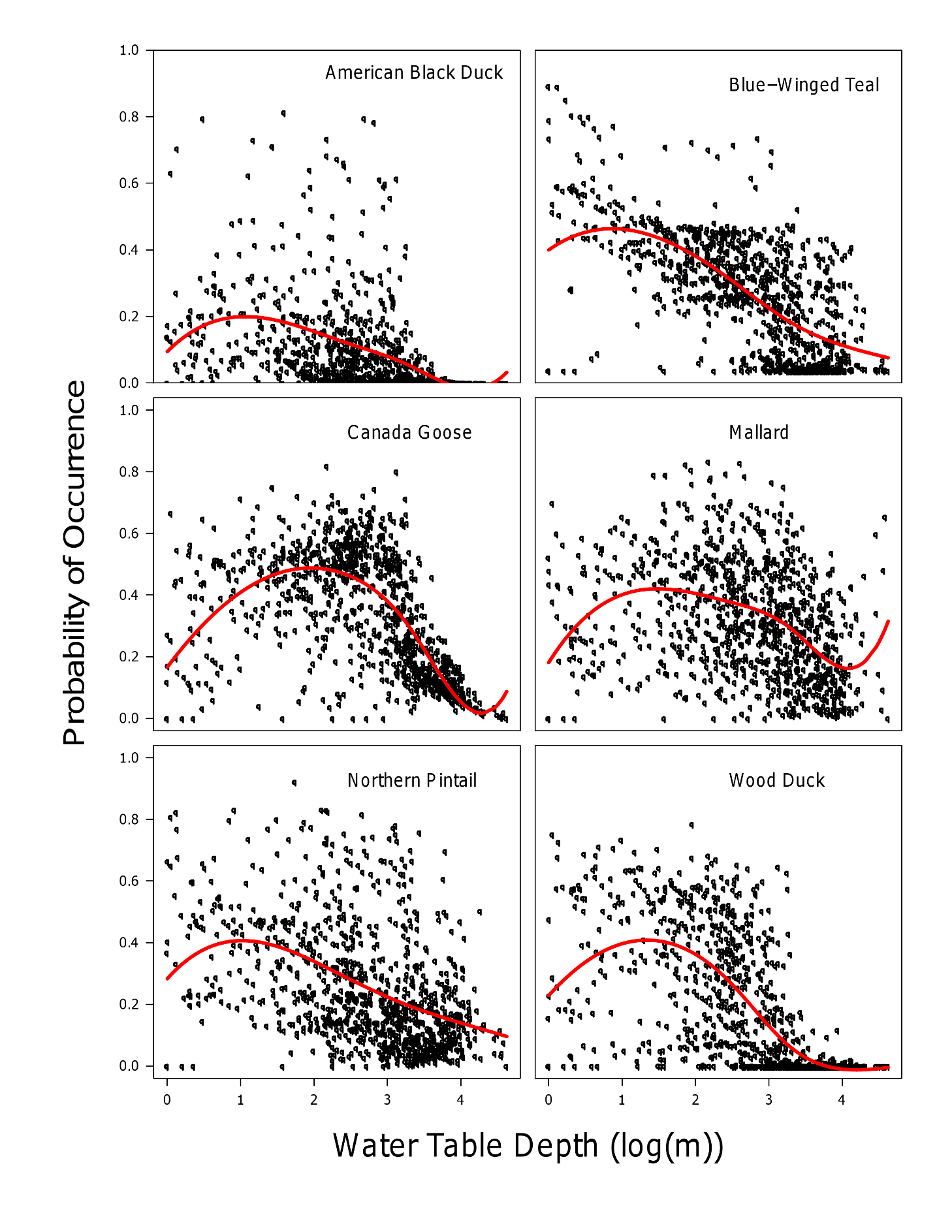

Supplement: Figure S6 — Plot of relationship between water table depth (m) and occurrence probability for species in spring. See Figure S5 for description. (TIF) [file pone.0030142.s006.tif]

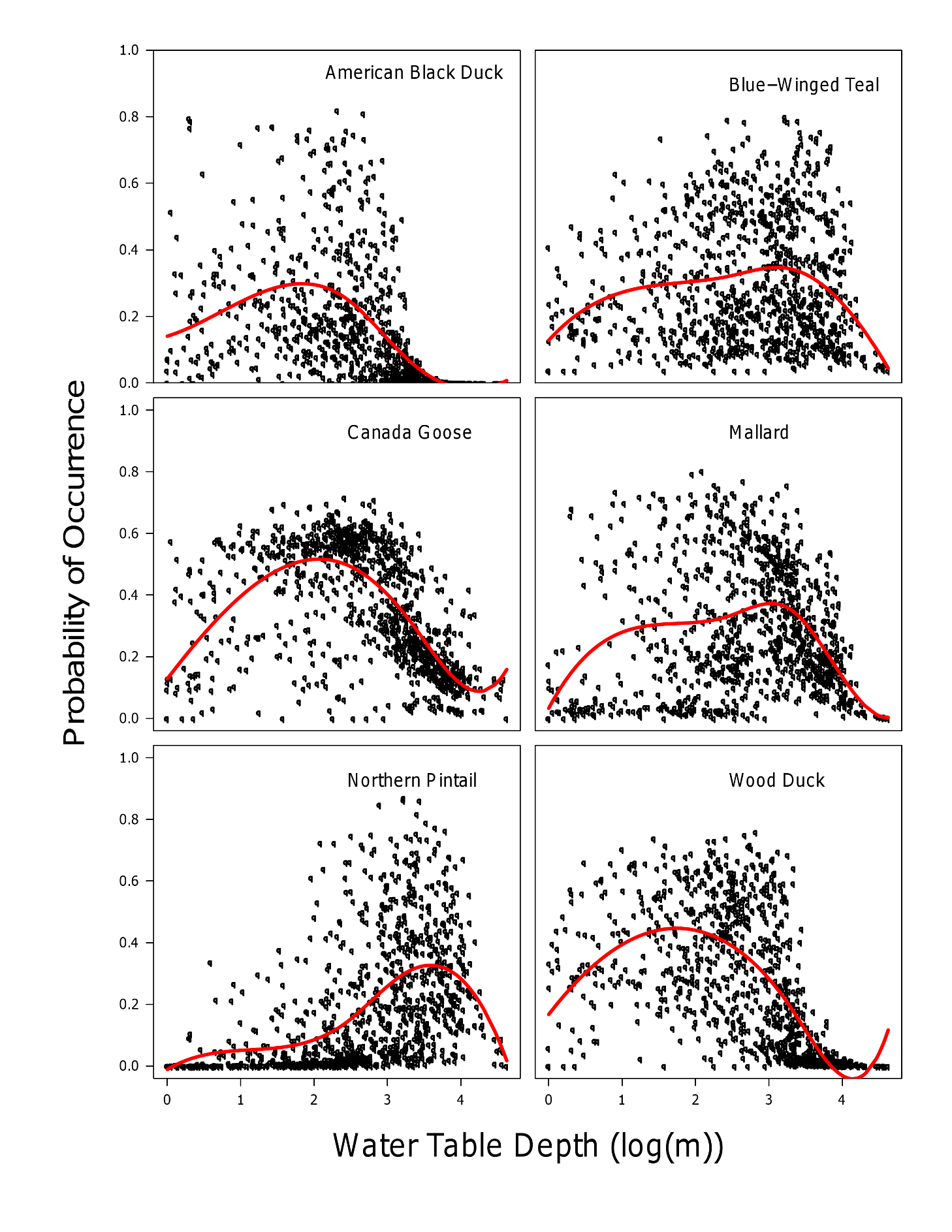

Supplement: Figure S7 — Plot of relationship between water table depth (m) and occurrence probability for species in summer. See Figure S5 for description. (TIF) [file pone.0030142.s007.tif]
